# Quantifying the evolutionary paths to endomembranes

**DOI:** 10.1016/j.celrep.2025.115533

**Published:** 2025-04-06

**Authors:** Paul E. Schavemaker, Michael Lynch

**Affiliations:** 1 Biodesign Center for Mechanisms of Evolution, Arizona State University, Tempe, AZ 85287, USA; 2 Lead contact

## Abstract

Eukaryotes exhibit a complex and dynamic internal meshwork of membranes—the endomembrane system—used to insert membrane proteins and ingest food. Verbal models explaining the origin of endomembranes abound, but quantitative considerations of fitness are lacking. Drawing on quantitative data on endomembranes allows for the derivation of two biologically grounded analytical models of endomembrane evolution that quantify organismal fitness: (1) vesicle-based uptake of small nutrient molecules, pinocytosis, and (2) vesicle-based insertion of membrane proteins, proto-endoplasmic reticulum. Surprisingly, pinocytosis of small-molecule nutrients does not provide a net fitness gain under biologically sensible parameter ranges, explaining why pinocytosis is primarily used for protein uptake in contemporary organisms. The proto-endoplasmic reticulum does provide net fitness gains, making it the more likely candidate for initiating the endomembrane system. With modifications, the approach developed here can be used more generally to understand the present-day endomembrane system and illuminate the origin of the eukaryotic cell.

## In brief

Schavemaker and Lynch derived functions that relate the cell biology of endomembranes to cellular fitness. Applied to the pinocytosis of small-molecule nutrients and the insertion of membrane proteins by a proto-endoplasmic reticulum, the proto-endoplasmic reticulum is revealed to be the more likely path to complex endomembranes in the origin of eukaryotes.

## Graphical Abstract

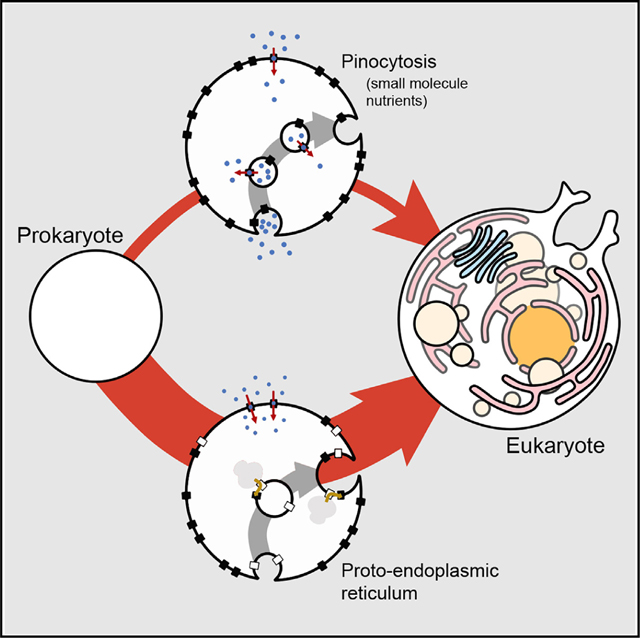

## INTRODUCTION

Eukaryotic cells are universally endowed with complex and dynamic internal membranes that are topologically separate from the plasma membrane. Examples are the endoplasmic reticulum (ER), Golgi apparatus, endosomes, and transport vesicles.^1^ Many verbal theories of the evolutionary origin of internal membranes, or endomembranes, have been proposed: originating as plasma membrane-attached tubules for protein excretion,^2^ as mitochondrial outer membrane-derived vesicles,^3^ or as a phagosome membrane involved in prey uptake.^4,5^ The inside-out model starts with tubules extending outward from what will become the nuclear compartment. These tubules swell into cytoplasmic compartments with the endomembranes forming at contact sites.^6^ Various other works discuss endomembrane evolution.^7–12^

Verbal theories can be supplemented by more quantitative theories of cellular traits to help decide between alternative hypotheses. Quantitative work has been performed on later stages of endomembrane evolution,^13^ on nuclear evolution,^14^ and on delayed bacterial digestion and mitochondria.^15^

Two dominant functions of endomembrane systems are (1) the uptake and processing of nutrients and (2) the production and insertion of membrane proteins. Simplified versions of these processes are candidates for intermediates in endomembrane evolution. For nutrient uptake the simplest system imaginable is vesicle cycling with transmembrane transport of small-molecule nutrients. For membrane-protein insertion, the simplest system is vesicle cycling with membrane-protein insertion happening on the vesicles. Both scenarios have endomembranes that are topologically distinct from the plasma membrane.

Key to the calculation of fitness is the observation that eukaryotes exhibit larger cell volumes than prokaryotes.^16^ It is likely that eukaryogenesis enabled this increase in volume or occurred in the context of it. As a cell increases its volume, the surface area available per unit volume diminishes. Some processes localized to the plasma membrane can be relegated to internal membranes, freeing up prime real estate for growth-limiting processes whose access to the external medium is essential. This selective pressure is specific to the (large-celled) eukaryotes, differing from selective pressures such as internal digestion of proteins that apply equally to (small-celled) prokaryotes.

Presented here are two alternative scenarios of early endomembrane evolution. In both, an intermediate evolutionary stage is considered in which vesicles emerge from the plasma membrane, spend some time in the cytoplasm, and fuse back into the plasma membrane. In the pinocytosis scenario, model 1, the vesicles transport small-molecule nutrients from their lumen into the cytoplasm. In the proto-ER scenario, model 2, the vesicles insert membrane proteins into their membrane and deliver these to the plasma membrane upon fusion. For both scenarios, the fitness of the derived, vesicle-containing state is calculated with respect to an ancestral state that lacks vesicles. A third scenario in which both cellular traits are combined is also considered. The results have implications for the understanding of eukaryogenesis and the cell biology of extant eukaryotes.

## RESULTS

### Outline of the pinocytosis model

The pinocytosis and proto-ER models utilize the same base function to calculate the net fitness, balancing (1) the gross fitness gain, (2) the energetic cost of a new trait, and (3) the area cost of plasma membrane occupancy by under-construction vesicles. These factors are combined in a fitness calculation that compares a derived state to an ancestral state (STAR Methods, Equation 1, Methods S1, and Equation S1).

Protein size and rate of conformational change are bounded. For membrane-embedded nutrient transporters, these basic physical facts imply that transport rates over a finite membrane cannot be infinite. Combined with another principle, that for expanding cells with invariant shape the cell volume grows faster than the cell surface area, it becomes obvious that when cells grow in volume over evolutionary time, nutrient demand will outstrip transport capacity. Pinocytosis of small-molecule nutrients could ameliorate this area-to-volume problem by internalizing some of the external nutrient-containing medium in vesicles and then transporting the nutrients from the vesicle lumens into the cytoplasm, increasing the effective cell surface area for nutrient transport. If the cell growth rate is limited by nutrient transport, this increased nutrient transport capacity increases growth rate and thereby fitness. We consider exclusively the pinocytosis of small-molecule nutrients. Pinocytosis of proteins and their internal digestion invokes different selective pressures and requires a more elaborate model.

**Figure 1.**
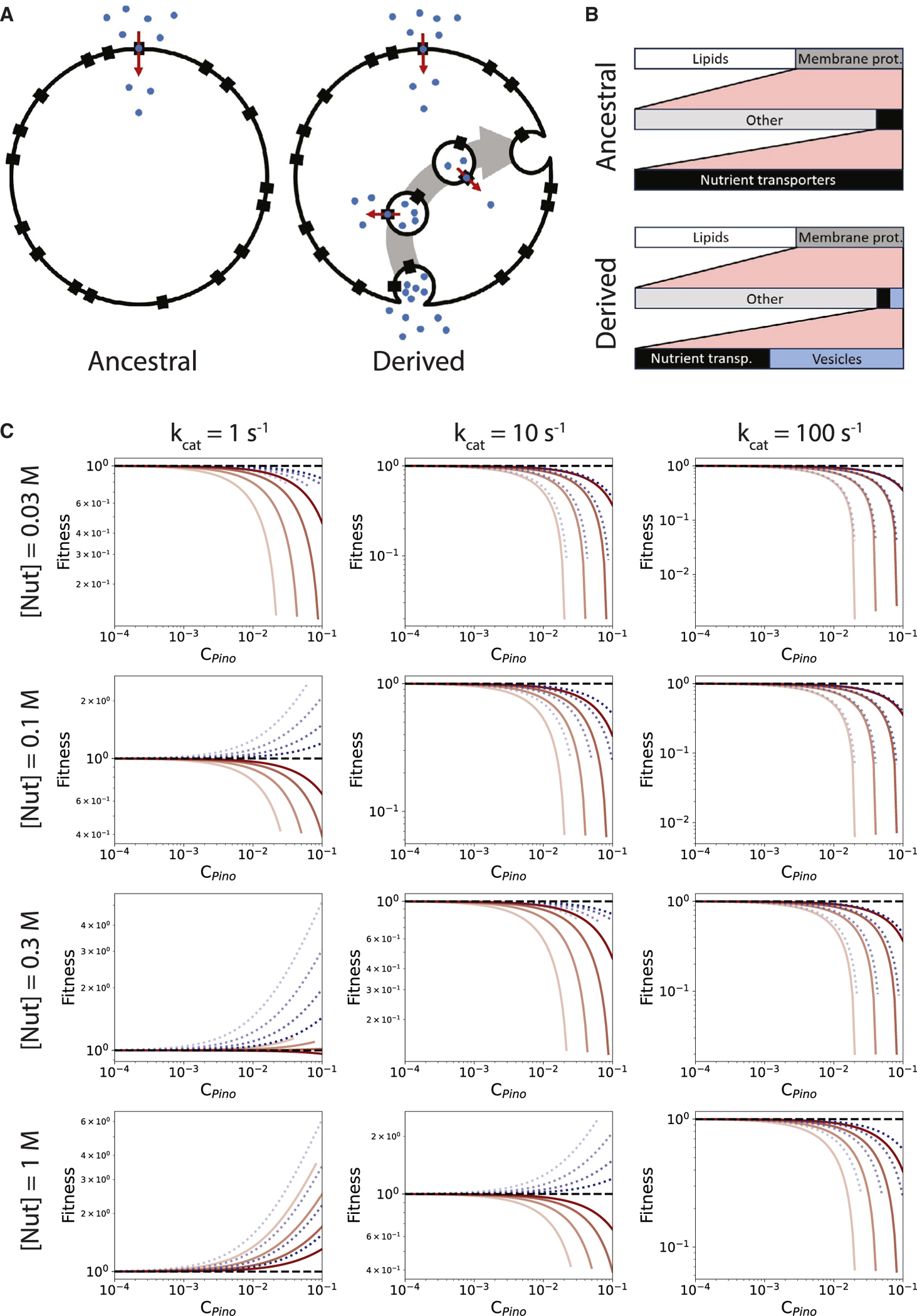
The pinocytosis model (A) Graphical representation of the pinocytosis model, showing the simple ancestral state and the more complex, pinocytic-vesicle carrying, derived state. Black rectangles, nutrient transporters; blue dots, nutrient molecules; red arrows, nutrient transport; gray arrow, vesicle progression. (B) Plasma membrane area occupancy in the ancestral and derived states. The length of the bars is proportional to the fractional occupancy in the plasma membrane. (C) Fitness of the derived, pinocytosis-exhibiting state as a function of the relative investment in pinocytosis (*C*_*pino*_), cell volume, transport rate (k_cat_), nutrient concentration, and vesicle size. Black dashed lines, ancestral fitness; red solid lines, vesicle radius 0.025 μm, blue dotted lines: vesicle radius 0.1 μm. Cell volume varies from 10, 10^2^, 10^3^, to 10^4^ μm^3^, with darker lines being the smaller volumes. Plots are cut off where the under-construction vesicles block the ancestral nutrient transporter area completely. Cell-division times are shown in Figure S2. Vesicle persistence times are listed in Tables S3 and S4.

The feasibility of pinocytosis as a precursor to the complex endomembrane system is evaluated by comparing two cellular states (Figure 1A): the ancestral state, a simple spherical cell that lacks internal membranes and that transports small-molecule nutrients over its plasma membrane using nutrient transporters, and the derived state, a slightly more complex spherical cell that in addition to having plasma membrane-based nutrient transport, produces vesicles from its plasma membrane that internalize nutrients. With the nutrients being transported from the vesicle lumens into the cytoplasm by vesicle-localized nutrient transporters. These considerations, in mathematical form, allow for the calculation of the cell-division times of both states (STAR Methods, Equations 2, 3, and 4; Methods S2, Equations S2, S3, S5, and S31).

The nutrient transport rate is limited by the area of the plasma membrane and by its composition (Figure 1B). In the model, the plasma membrane contains 60% lipids by area, and 40% protein.^17^ Of the protein area, 90% is assumed to do something other than nutrient transport. In the ancestral state, the remaining 10% of the protein area is used for nutrient transport. In the derived state, this 10% is used for both nutrient transport and producing vesicles.

The overall nutrient transport rate over the plasma membrane and over the vesicle membranes depends on the number of vesicles present at each moment. The vesicle number is calculated from the investment in pinocytosis, *C*_*pino*_, which derives from the membrane cost^18,19^ and the costs of the 16 proteins that construct the 50-nm-diameter vesicles (Figure S1, Table S1, and Methods S2).^20^

### Pinocytosis reduces fitness at low nutrient concentrations

**Figure 2.**
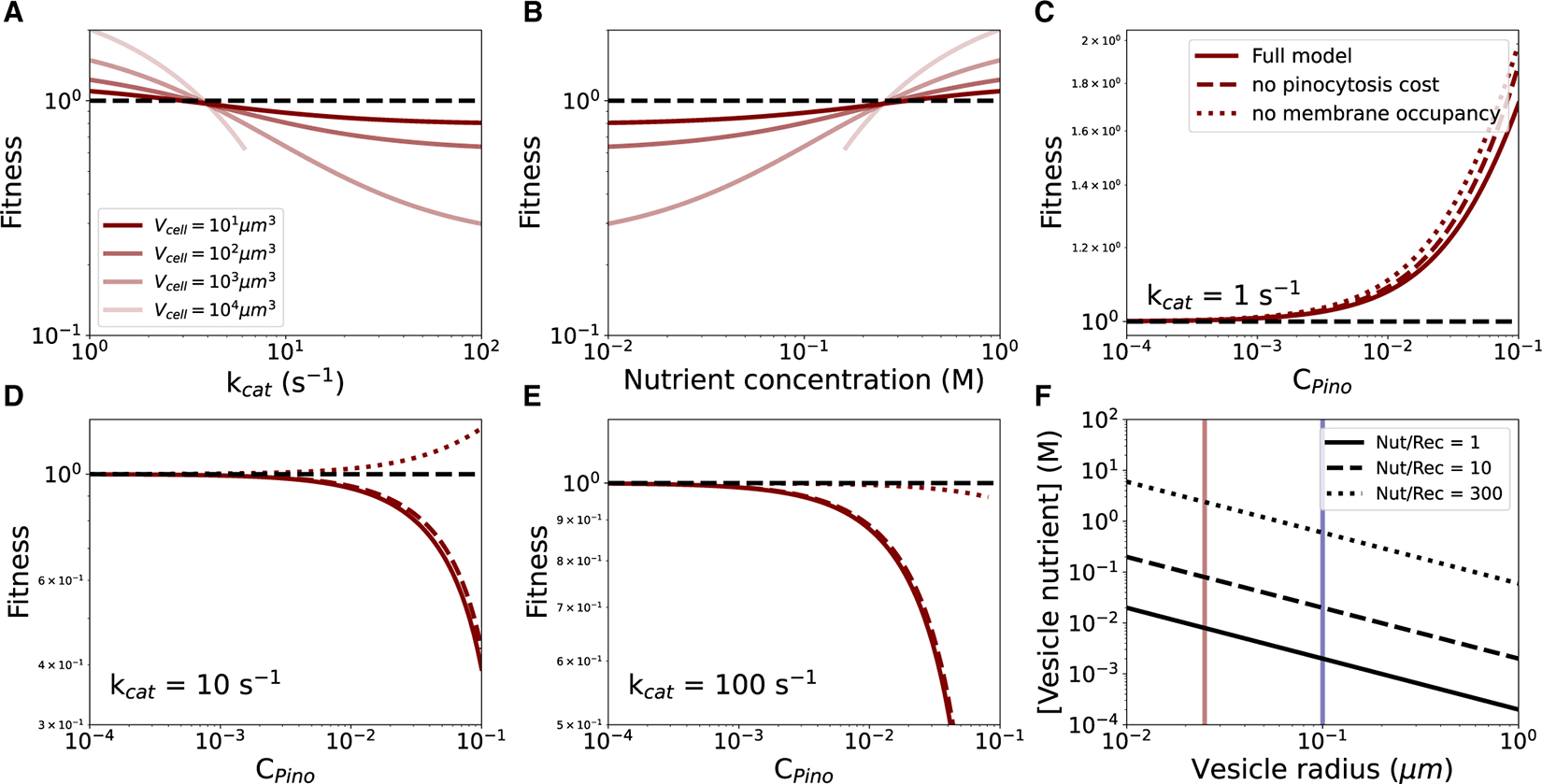
Analysis of the contributions to pinocytosis fitness (A) Fitness versus the nutrient transport rate parameter k_cat_. Vesicle radius, 0.025 μm; relative pinocytosis cost, 0.03; nutrient concentration, 1 M; V_cell_, cell volume. (B) Fitness versus the external nutrient concentration. Vesicle radius, 0.025 μm; relative pinocytosis cost, 0.03; k_cat_, 1 s^−1^. (A and B) For the cell volume of 10^4^ μm^3^, the plot is cut off when the under-construction vesicles completely block the nutrient-transporter area on the plasma membrane. (C–E) Comparison of the fitness calculated from the full model to the fitness calculated in the absence of pinocytosis cost or plasma membrane occupancy by under-construction vesicles. Cell volume, 10^2^ μm^3^; nutrient concentration, 1 M. (F) The effect of vesicle-localized receptors on internal vesicle nutrient concentration. Vertical lines show the vesicle radii used in the pinocytosis model (red line, 0.025 μm; blue line, 0.1 μm). Nut/Rec, number of nutrient molecules able to bind a single receptor.

Pinocytosis can only be advantageous if nutrient transport over the vesicle membranes can outpace nutrient transport over the plasma membrane. As the investment in pinocytosis increases, the number of vesicles produced during the cell cycle increases, and in the ideal case, this causes the cell-division time to decline and the fitness to increase (relative to the ancestor). Figure 1C (solid red lines) reveals the fitness at varying cell volume, external nutrient concentration, and turnover number of the nutrient transporter. Only for turnover numbers of 1 s^−1^ and for nutrient concentration of 0.3 M and above does fitness improve in the derived, pinocytosis-exhibiting state. Larger cell volumes improve the effectiveness of pinocytosis. A higher-resolution view of the effect of nutrient concentration and turnover number on fitness leads to the same conclusions (Figures 2A and 2B). The turnover number for glucose transport is 100 s^−1^,^21,22,23^ which means that a cell living on glucose would never evolve pinocytosis to increase nutrient uptake rate, as concentrations of 0.3 M and above are presumably rare in nature.

A reason for the failure of pinocytosis for increasing nutrient uptake can be gleaned from examining the two main detractors of fitness: (1) the cost of maintaining and producing vesicles and (2) the plasma membrane area occupied by under-construction vesicles. Figures 2C–2E show that the largest fitness penalty is the membrane occupation of under-construction vesicles. This means that anything that shortens the residence time or reduces area occupancy of under-construction vesicles on the plasma membrane could have a major effect on the possibility of the evolution of pinocytosis.

An increase in vesicle radius reduces the area occupancy per vesicle-localized nutrient molecule, so large (200 nm diameter) vesicles work better than small (50 nm) vesicles (Figure 1C), but this still depends on low turnover numbers for nutrient transport and high external nutrient concentration. Concentration of nutrient molecules by receptors could in principle improve the effectiveness of pinocytosis at low external nutrient concentrations. However, Figure 2F reveals that this would only work if (excessively) many nutrients bind each available receptor and receptors occupy most of the vesicle membrane (see Methods S2, “Receptors for concentrating nutrient molecules in vesicles”). Thus, pinocytosis for small-molecule nutrient uptake is unlikely to be a precursor to the endomembrane system and appears to be an unproductive strategy for nutrient uptake in extant organisms.

### Outline of the proto-ER model

If the cell volume is increased, then the surface area-to-volume ratio is reduced. This makes it advantageous to internalize membrane-localized processes that do not require access to the cell surface. The insertion of membrane proteins requires membrane but not direct access to the cell surface. The Sec translocases that facilitate membrane-protein insertion and the associated ribosomes can thus be housed on internal membranes if a mechanism for membrane protein delivery to the plasma membrane exists.^24,25^

**Figure 3.**
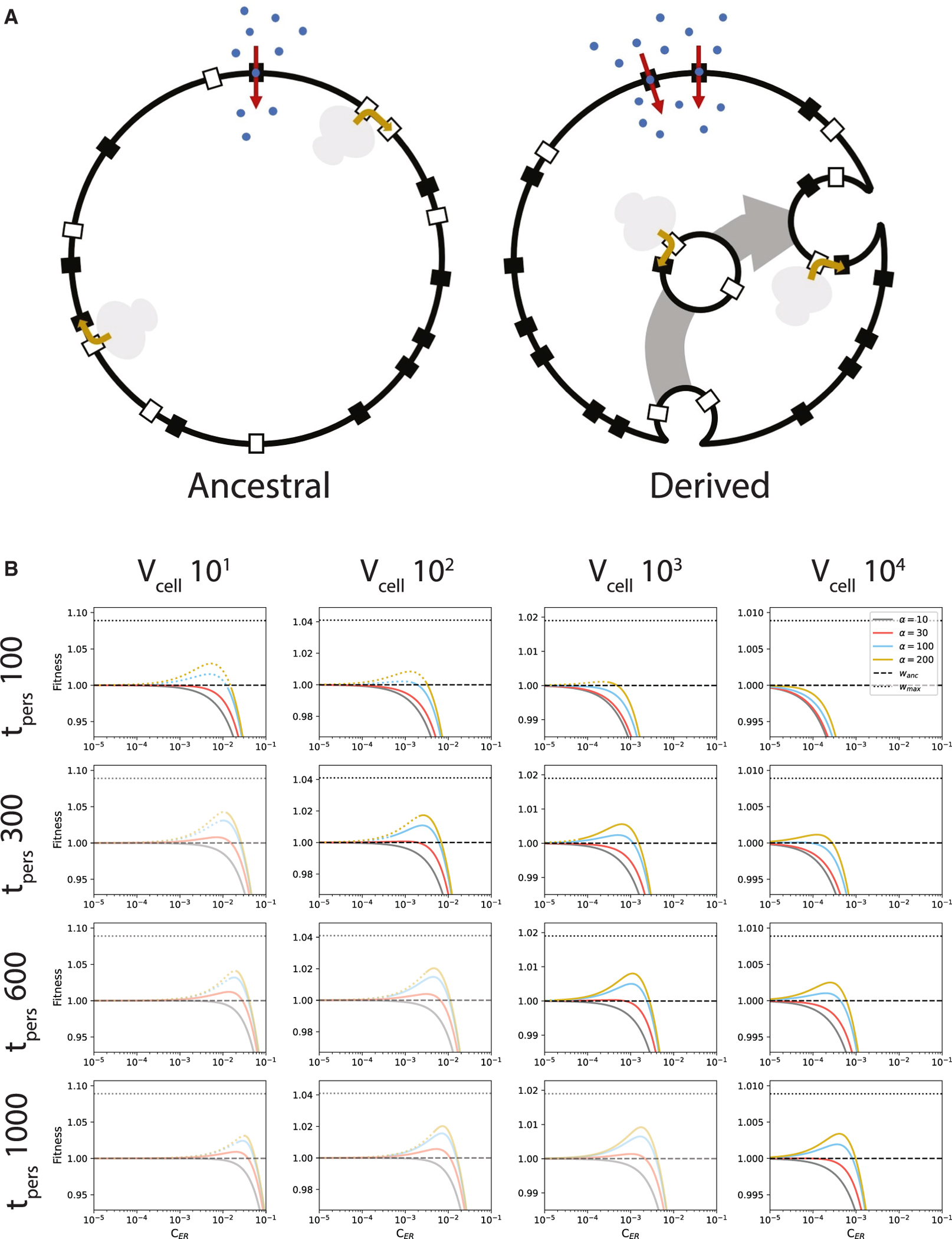
The proto-ER model (A) Graphical representation of the proto-ER model, showing the simple ancestral state and the more complex, vesicle-exhibiting derived state. Black rectangles, nutrient transporters; white rectangles, Sec translocases; gray bilobed patches, ribosomes; blue dots, nutrient molecules; red arrows, nutrient transport; golden arrows, membrane-protein insertion; gray arrow: vesicle progression. (B) The fitness of the derived state as a function of the relative cost of the proto-ER. The insertion time for individual membrane proteins (*t*_*ins*_) is 20 s; cell volumes (*V*_*cell*_) in μm^3^; vesicle persistence times (*t*_*pers*_) in seconds. The parameter α shows the concentration factor for Sec translocases from plasma membrane into vesicles. Dotted colored lines show where the model breaks down due to ribosome packing on the vesicle surface. Dashed black lines, fitness of the ancestor; dotted black lines, maximal attainable fitness. Faded plots indicate the parameter combinations for which *t*_*pers*_/*t*_*d*_ > 0.1. To derive the model, it was assumed that *t*_*pers*_/*t*_*d*_ ≪ 1 (Methods S3, Equations S41 and S42). Cell division times are shown in Figure S3.

The proto-ER model compares the fitness of a simple ancestral cell, lacking internal membranes, to a somewhat more complex cell that houses vesicles in the cytoplasm (Figure 3A). These vesicles are produced at the plasma membrane and concentrate Sec translocases in their membranes. These Sec translocases insert membrane proteins, causing the vesicles to grow (lipids are assumed to insert in tandem with membrane proteins). After persisting in the cytoplasm for some time, these vesicles fuse back into the plasma membrane to deliver their cargo.

This cycling of vesicles permanently internalizes a fraction of the Sec translocases, freeing up area on the plasma membrane for more nutrient transporters. It is this increase in the number of nutrient transporters that via an increased total nutrient import rate and a shorter cell-division time that (potentially) increases the fitness of the derived state. The cell-division times are calculated as described in the STAR Methods (Equations 2, 3, and 4) and in Methods S2 and S3 (Equations S2, S3, S5, and S31). The energetic cost of the proto-ER is the same as for pinocytosis, except that the cytoplasmic vesicles grow over time and an average cost is used (Methods S2 and S3, Equations S36–S45).

### The proto-ER improves fitness

As the proto-ER is introduced, the concentration of Sec translocon in the plasma membrane is reduced, expanding the area available to nutrient transporters. An increase in the number of nutrient transporters decreases the cell-division time. Fitness can be determined by combining the cell-division times of the ancestral and derived states and depends on the relative construction cost of the proto-ER (Equation 1; parameter values in Table S2).

**Figure 4.**
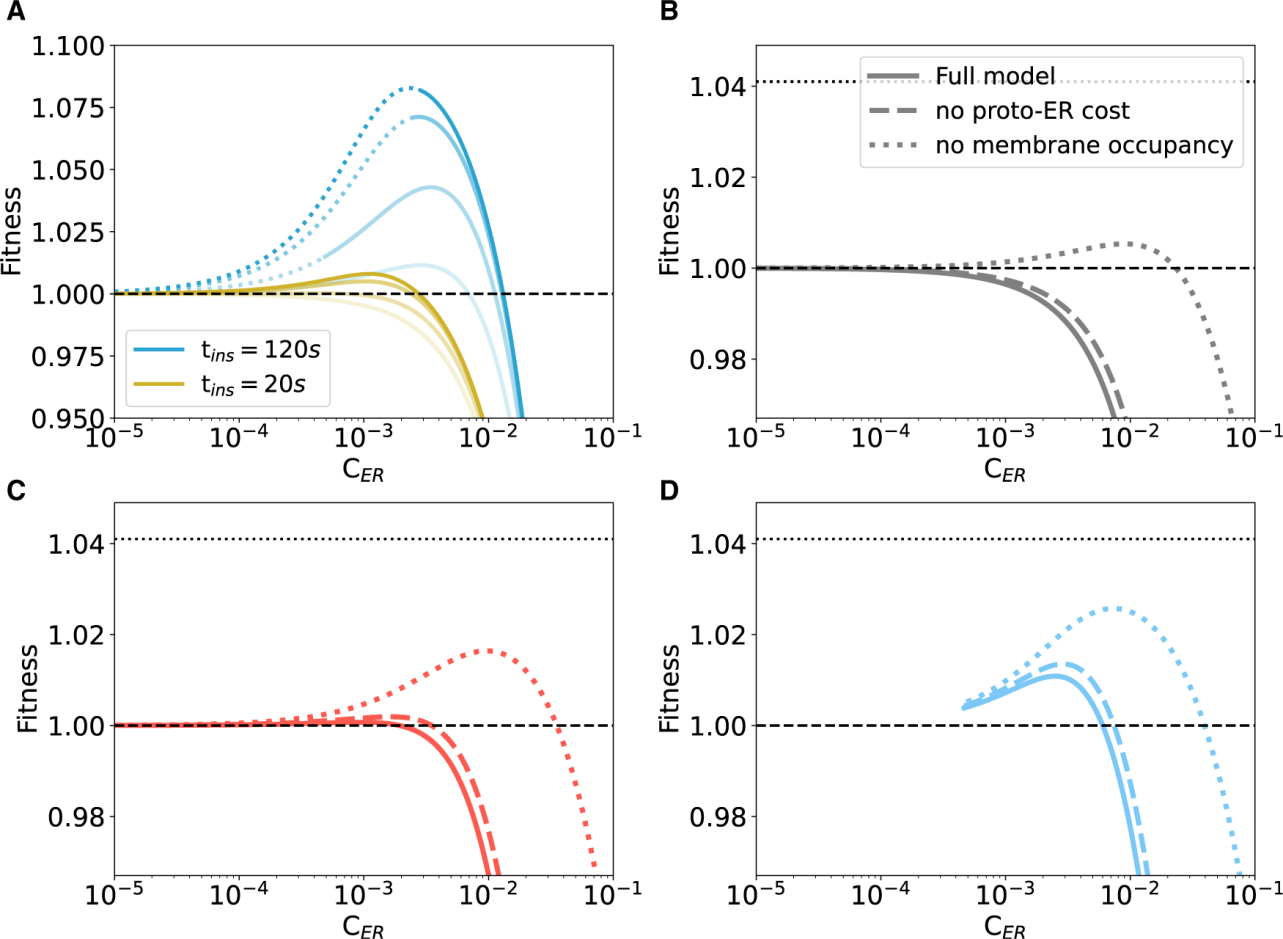
Analysis of the contributions to proto-ER fitness (A) Fitness of the derived state for two membrane protein insertion times, *t*_*ins*_. The different shades correspond to different concentration factors α, from light to dark 10, 30, 100, and 200. Black dashed line, ancestral fitness; cell volume, 10^3^ μm^3^; vesicle persistence time, 600 s. Fitness and cell division times over the full parameter value range are shown in Figures S4 and S5. (B–D) Comparison of the fitness calculated from the full model to the fitness calculated in the absence of proto-ER cost or plasma membrane occupancy by under-construction vesicles. Black dashed line, ancestral fitness; black dotted line, maximal attainable fitness. The lines in (D) start at the ribosome limit. Cell volume, 10^2^ μm^3^; vesicle persistence time, 300 s; membrane protein insertion time, 20 s. (B) α = 10, (C) α = 30, (D) α = 100.

Examining parameter space reveals that the proto-ER can improve fitness at larger Sec translocon concentration factors and longer vesicle persistence times (Figure 3B). These results were obtained with a membrane protein insertion time, *t*_*ins*_, of 20 s. This insertion time is an estimate derived from the 20 amino acids (aa)/s protein elongation rate in *Escherichia coli*,^26^ the average protein length of somewhat over 300 aa,^27^ and the knowledge that membrane proteins are inserted co-translationally.^24,25^ To account for a potential slowing of protein synthesis during membrane-protein insertion, and to account for different protein-elongation rates in different species (the lower limit being 3 aa/s^26,28^), the insertion time was extended to 120 s, leading to higher fitness (Figures 4A and S4).

Introducing a proto-ER comes with two major costs: (1) the energetic costs of producing and maintaining the vesicles and (2) the cost of occupying plasma membrane area by under-construction vesicles. Removing these costs from the models (Figures 4B–4D) shows that the plasma membrane area occupation by under-construction vesicles constitutes the largest penalty, as it does for the pinocytosis model.

Vesicles of the proto-ER carry nutrient molecules by default, and combining the pinocytosis mechanism with that of the proto-ER may yield a fitness boost. An extra nutrient influx by transporters in the vesicles would come for free as the vesicles already exist. However, the combined model (Methods S3 and Equation S61) shows no additional benefit over the proto-ER alone (Figure S6). Despite this lack of an additional benefit, pinocytosis may evolve in concert with the proto-ER simply by nutrient transporters being incompletely excluded from the vesicles.

## DISCUSSION

### An intuitive explanation for the pinocytosis and proto-ER models

A patch of plasma membrane area can only be used by one process at a time. For pinocytosis to work, the benefit of producing a vesicle must outstrip the benefit of using that same patch of plasma membrane area for nutrient transport directly. The maximal number of nutrient molecules that can be imported into the cytoplasm by a single vesicle is equal to the number of nutrient molecules present in the vesicle just after it is formed. And it is this number that needs to exceed the number of nutrients being transported directly by this patch of plasma membrane if it had been occupied by nutrient transporters. This explains why larger vesicles and higher external nutrient concentrations work better: they provide more nutrient molecules per unit plasma membrane area. This also explains why a slower nutrient transporter works in favor of pinocytosis—it lowers the threshold number of nutrient molecules that are required to be present in the pinocytic vesicles at the start.

The proto-ER faces a similar trade-off. The benefit of producing a vesicle needs to outstrip the benefit of using that same patch of membrane area for membrane protein insertion. However, the proto-ER has two crucial advantages over pinocytosis. First, the number of membrane protein insertions that a single vesicle can accomplish increases with the time the vesicle spends in the cytoplasm. This is different from the pinocytosis model, where no further gains can be made once the vesicle is emptied. The second advantage is that the extra nutrient transporters are added to the plasma membrane where the pool of nutrients is effectively infinite. In the pinocytosis model, in contrast, the extra nutrient transporters are added on the vesicles where the pool of nutrient molecules is small. The proto-ER model therefore succeeds under sensible biological parameters, whereas the pinocytosis model works only under excessively high nutrient concentrations. Thus, an evolutionary path toward complex endomembranes that passes through a proto-ER is more probable.

If the pinocytosis model does not work, why do cells perform pinocytosis? Most descriptions of pinocytosis feature proteins or other large particles as the target of pinocytosis,^29–37^ the exception being sucrose uptake in large vesicles by *Amoeba proteus*.^38,39^ In contrast, fungi digest their food externally and take up small molecules, but uptake is facilitated by plasma membrane localized transporters,^40^ not pinocytosis. Thus, pinocytosis of small nutrient molecules is unlikely to be a competitive evolutionary strategy, but pinocytosis of macromolecules probably is.

### Implications for cell biology and evolution

Cell biological transitions are the result of thousands of mutations and fixations. To guide our understanding, we require a coarse-grained understanding of the fitness contributions associated with intermediate states along evolutionary paths. Here, we complement coarse-grained verbal theories^2–12^ with a quantitative approach. Pinocytosis of small molecules and a proto-ER can be understood via similar quantitative models, with broad cell biological implications because many other traits also depend on the transfer of vesicles.^1,2,41–44^ Ultimately, this will allow us to address the deeper question of why ubiquitous vesicle transport exists in the first place, and why evolution decided against putting a dedicated Sec translocase on every internal compartment.

An alternative to our vesicle models for solving area-to-volume issues are cell elongation or membrane ruffle formation. However, these face trade-offs (e.g., osmotic effects) that need to be examined quantitatively before they can be judged as feasible alternatives. The vesicle model could also work in tandem with these other solutions as these reach limiting trade-offs. An alternative selective pressure for pinocytic vesicle evolution, internal digestion of proteins, would not compete with these other mechanisms, but it does not explain why endomembranes are mostly confined to large cells (eukaryotes) as internal digestion would benefit small cells equally.

Complexity of vesicle formation and fusion necessitate gradual emergence, with initial iterations working slower and less efficiently^45^ and exhibiting only limited gains in fitness. Precursors of soluble *N*-ethylmaleimide-sensitive factor activating protein receptors (SNAREs) and other fusion proteins as well as coat proteins and actin, used in eukaryotic vesicle formation, are present in Archaea,^46–48^ as are precursors to key guanosine triphosphatases that regulate membrane trafficking in eukaryotes.^49,50^ Future evolutionary models will have to incorporate the capacities of this repertoire and how it changed over time into quantitative models of fitness. Similar considerations are associated with the transition between archaeal and eukaryotic lipids,^3^ which may affect the ease of manipulating membranes and therefore the parameters that feature in the models.

The fitness-function approach could be applied to other scenarios of endomembrane origin. It has been proposed that mitochondria may have initiated the formation of the complex endomembrane system.^51^ The influence of mitochondria on endomembrane evolution and vice versa could be investigated by combining results obtained here with a previous modeling study on mitochondria,^16^ or from fitness functions for outer mitochondrial membrane-derived endomembranes.^51^ The inside-out model^6^ of endomembrane evolution proposes a drastically different sequence of events from ours, but it also includes a vesicle-trafficking step to which our fitness-function approach could apply, albeit with different selective pressures. Similar considerations hold for phagocytosis-first scenarios^5^ and endomembrane-based food sequestration for use in times of scarcity.^15^

### Limitations of the study

In the present study, two selective pressures were examined, comparing just two cellular states with primitive endomembranes working in isolation. A more comprehensive understanding of eukaryogenesis and the cell biology of endomembranes would require modeling alternative selective pressures such as internal digestion of proteins, phagocytosis, the inside-out model, and outer mitochondrial membrane vesicles. In addition, we would need to introduce more cellular states comprising the gradual emergence or recruitment of critical molecular machinery such as actin, coat proteins, SNAREs, and more. Eukaryogenesis also involves mitochondria and cytoskeleton, and their interaction with endomembranes needs to be examined. Finally, to reduce the number of assumptions made in deriving the model, the fitness function needs to be extended to account for other factors than cell division time.

### Conclusion

Two competing models for the early evolution of endomembranes were derived in which fitness is calculated explicitly from cell biological traits. The pinocytosis model, for the uptake of small-molecule nutrients, fails to improve fitness under plausible nutrient concentrations. The proto-ER model does not suffer from this problem and is the more likely transitional state from simple cells, which lack internal membranes, to modern eukaryotic cells that sport a manifold endomembrane system. That the uptake of small molecules by pinocytosis is not favored by natural selection is consistent with and explains the distribution and nature of pinocytosis across the tree of life. Extensions of the approach presented here will improve the understanding of eukaryotic origins and the cell biology of present-day organisms.

## RESOURCE AVAILABILITY

### Lead contact

Requests for further information should be directed to Paul E. Schavemaker (pschavem@asu.edu).

### Materials availability

No materials were generated in this study.

### Data and code availability

- This paper analyzes existing, publicly available data cited in the main text.
- This paper does not report original code.
- Any additional information required to reanalyze the results presented in this paper is available from the lead contact upon request.

## STAR★METHODS

### METHOD DETAILS

#### Defining a fitness function

Determining the fitness change upon the introduction of a new cellular trait involves balancing the gains with the energetic (and other) costs that result from the investment in proteins, membranes, etc. Following Lynch and Trickovic,^53^ it is assumed that the incorporation of additional resources by the cell, delays the onset of cell division. This causes a reduction in fitness that is approximated as the relative energetic cost of the new trait, $C_{\mathrm{trait}}$, expressed as the ratio between the energetic cost of the new trait divided by the cell budget. Both the trait cost and cell budget are expressed in units of ATP and include opportunity costs.^54–56^ The benefit in both the pinocytosis and proto-endoplasmic reticulum mechanisms is an increase in the transport rate of small-molecule nutrients, which reduces cell division time. From these considerations, and comparing the cell division time of an ancestral state to that of a derived state we define the following fitness function:

(Equation 1) $$\mathrm{Fitness}=\frac{t_{d,\mathrm{anc}}}{t_{d,der}}=\frac{t_{d,\mathrm{anc}}}{{t_{d,\mathrm{der}}}^{*}\left(1\;+\;C_{\mathrm{trait}}\right)}$$

Here, $t_{d,\mathrm{anc}}$ is the cell division time of the ancestral state, $t_{d,\mathrm{der}}$ is the cell division time of the derived state, ${t_{d,\mathrm{der}}}^{*}$ is the cell division time of the derived state at zero energetic cost, and $C_{\mathrm{trait}}$ is the relative cost of the new trait (either pinocytosis or the proto-endoplasmic reticulum).

#### Cell division times for pinocytosis

The cell division time of the ancestral state is calculated from the nutrient requirement per unit cell volume, $N_{\mathrm{nutV}}$, the cell volume at birth, $V_{\mathrm{cell},0}$, and the whole-cell nutrient import rate, $v_{\mathrm{trans},PM}$ (Methods S2 and Equation S2):

(Equation 2) $$t_{d,\mathrm{anc}}=\frac{N_{\mathrm{nutV}}V_{\mathrm{cell},0}}{V_{\mathrm{trans},PM}}$$

The numerator gives the nutrient requirement for producing a new cell and it is assumed that the cell division time is nutrient limited. The whole cell nutrient import rate is given by:

(Equation 3) $$v_{\mathrm{trans},PM}=k_{\mathrm{cat}}\frac{\left[nut\right]}{K_{M}\;+\;[nut]}\bar{N_{\mathrm{trans}}}$$

Here, $k_{\mathrm{cat}}$ is the turnover number, [nut] is the external nutrient concentration, $K_{M}$ is the Michaelis constant, and $\bar{N_{\mathrm{trans}}}$ is the number of nutrient transporters averaged over the cell cycle.

For the derived state, the cell division time is calculated similarly but includes the transport rate of nutrients over the combined vesicle membranes, $v_{\mathrm{trans},\mathrm{pino}}$, and discounts the contribution of plasma membrane nutrient transport to provide surface area for vesicle production, $v_{\mathrm{trans,PM}}$ (Methods S2 and Equation S5):

(Equation 4) $$t_{d,\mathrm{pino}}=\frac{N_{\mathrm{nutV}}V_{\mathrm{cell,}0}\left(1\;+\;C_{\mathrm{pino}}\right)}{V_{\mathrm{trans,PM}}*\;+\;V_{\mathrm{trans,pino}}}$$

Here, $C_{\mathrm{pino}}$ is the relative energetic cost of pinocytosis. Parameter values for the calculations are listed in Table S2.

#### Cell division times for the proto-ER

For the proto-endoplasmic reticulum, the fitness is calculated using Equation 1, the same as for pinocytosis. The difference resides in the calculation of the cell division times. For the proto-endoplasmic reticulum the cell division time of the ancestor is calculated with Equations 2 and 3, but with an $\bar{N_{\mathrm{trans}}}$ that depends on the number of Sec translocons (Methods S3 and Equation S30). The cell division time of the derived state, $t_{d,ER}$, is given by (Methods S3 and Equation S31):

(Equation 5) $$t_{d,ER}=\frac{N_{\mathrm{nutV}}V_{\mathrm{cell},0}\left(1\;+\;C_{ER}\right)}{k_{\mathrm{cat}}\frac{\left[nut\right]}{K_{M}\;+\;[nut]}(\bar{N_{\mathrm{trans}}}\;-\;(\bar{N_{\mathrm{SecPM}}}\;-\;\bar{N_{\mathrm{Sec,anc}}})\;-\;\bar{N_{\mathrm{occ}}})}$$

Here, $C_{ER}$ is the relative energetic cost of the proto-endoplasmic reticulum, $\bar{N_{SecPM}}$ is the number of Sec translocases in the plasma membrane averaged over the cell cycle, $\bar{N_{\mathrm{Sec,anc}}}$ is the average number of Sec translocases in the plasma membrane of the ancestor, and $\bar{N_{\mathrm{occ}}}$ is the average number of nutrient transporters displaced by under-construction vesicles. The combination – ($\bar{N_{\mathrm{SecPM}}}-\bar{N_{\mathrm{Sec,anc}}}$) is the gain in the number of nutrient transporters on the plasma membrane. The equations for the average numbers of nutrient transporters and Sec translocases^57^ are worked out in the Methods S2 and S3 (Equations S18, S30, S32, and S34). Parameter values for the calculations are listed in Table S2.

All calculations were performed in Mathematica and the plots were generated with Matplotlib in Python.

### QUANTIFICATION AND ANALYSIS

There are no statistical analyses to include in this study.

## Supplementary Material

1

## Figures and Tables

**KEY RESOURCES TABLE:** 

REAGENT or RESOURCE	SOURCE	IDENTIFIER

Software and algorithms		

Wolfram Mathematica	Wolfram Research	https://www.wolfram.com/mathematica/
Python 3.8.2	Python Software Foundation	https://www.python.org/
Matplotlib	Hunter^52^	https://matplotlib.org/

## Highlights

- Mathematical functions couple the cell biology of endomembranes to fitness
- Pinocytosis of small-molecule nutrients is ineffective
- Proto-endoplasmic reticulum could be an intermediate to complex endomembranes
- Fitness functions of cellular traits help us to understand eukaryogenesis
